# SCC*mec*Finder, a Web-Based Tool for Typing of Staphylococcal Cassette Chromosome *mec* in *Staphylococcus aureus* Using Whole-Genome Sequence Data

**DOI:** 10.1128/mSphere.00612-17

**Published:** 2018-02-14

**Authors:** Hülya Kaya, Henrik Hasman, Jesper Larsen, Marc Stegger, Thor Bech Johannesen, Rosa Lundbye Allesøe, Camilla Koldbæk Lemvigh, Frank Møller Aarestrup, Ole Lund, Anders Rhod Larsen

**Affiliations:** a Department of Bacteria, Parasites and Fungi, Statens Serum Institut, Copenhagen, Denmark; b Research Group for Genomic Epidemiology, National Food Institute, Technical University of Denmark, Kgs Lyngby, Denmark; c Center for Biological Sequence Analysis, Department of Bioinformatics, Technical University of Denmark, Kgs Lyngby, Denmark; U.S. Centers for Disease Control and Prevention

**Keywords:** MRSA, SCC*mec*, bioinformatics, *mecA*, *mecC*, typing

## Abstract

Typing of methicillin-resistant *Staphylococcus aureus* (MRSA) is important in infection control and surveillance. The current nomenclature of MRSA includes the genetic background of the *S. aureus* strain determined by multilocus sequence typing (MLST) or equivalent methods like *spa* typing and typing of the mobile genetic element staphylococcal cassette chromosome *mec* (SCC*mec*), which carries the *mecA* or *mecC* gene. Whereas MLST and *spa* typing are relatively simple, typing of SCC*mec* is less trivial because of its heterogeneity. Whole-genome sequencing (WGS) provides the essential data for typing of the genetic background and SCC*mec*, but so far, no bioinformatic tools for SCC*mec* typing have been available. Here, we report the development and evaluation of SCC*mec*Finder for characterization of the SCC*mec* element from *S. aureus* WGS data. SCC*mec*Finder is able to identify all SCC*mec* element types, designated I to XIII, with subtyping of SCC*mec* types IV (2B) and V (5C2). SCC*mec* elements are characterized by two different gene prediction approaches to achieve correct annotation, a Basic Local Alignment Search Tool (BLAST)-based approach and a *k*-mer-based approach. Evaluation of SCC*mec*Finder by using a diverse collection of clinical isolates (*n* = 93) showed a high typeability level of 96.7%, which increased to 98.9% upon modification of the default settings. In conclusion, SCC*mec*Finder can be an alternative to more laborious SCC*mec* typing methods and is freely available at https://cge.cbs.dtu.dk/services/SCCmecFinder.

**IMPORTANCE** SCC*mec* in MRSA is acknowledged to be of importance not only because it contains the *mecA* or *mecC* gene but also for staphylococcal adaptation to different environments, e.g., in hospitals, the community, and livestock. Typing of SCC*mec* by PCR techniques has, because of its heterogeneity, been challenging, and whole-genome sequencing has only partially solved this since no good bioinformatic tools have been available. In this article, we describe the development of a new bioinformatic tool, SCC*mec*Finder, that includes most of the needs for infection control professionals and researchers regarding the interpretation of SCC*mec* elements. The software detects all of the SCC*mec* elements accepted by the International Working Group on the Classification of Staphylococcal Cassette Chromosome Elements, and users will be prompted if diverging and potential new elements are uploaded. Furthermore, SCC*mec*Finder will be curated and updated as new elements are found and it is easy to use and freely accessible.

## Abstract (precis)

SCC*mec* in MRSA is acknowledged to be of importance not only because it contains the *mecA* or *mecC* gene but also for staphylococcal adaptation to different environments, e.g., in hospitals, the community, and livestock. Typing of SCC*mec* by PCR techniques has, because of its heterogeneity, been challenging, and whole-genome sequencing has only partially solved this since no good bioinformatic tools have been available. In this article, we describe the development of a new bioinformatic tool, SCC*mec*Finder, that includes most of the needs for infection control professionals and researchers regarding the interpretation of SCC*mec* elements. The software detects all of the SCC*mec* elements accepted by the International Working Group on the Classification of Staphylococcal Cassette Chromosome Elements, and users will be prompted if diverging and potential new elements are uploaded. Furthermore, SCC*mec*Finder will be curated and updated as new elements are found and it is easy to use and freely accessible.

## INTRODUCTION

The presence of methicillin-resistant *Staphylococcus aureus* (MRSA) imposes a significant burden on the public health care system, where accurate molecular typing is essential for infection control and surveillance of MRSA. The current standard MRSA nomenclature includes identification of the chromosomal background, annotated by the multilocus sequence type (ST) or clonal complex (CC) and the type of staphylococcal cassette chromosome *mec* (SCC*mec*) element (indicated by roman numerals I to XIII), sometimes with the addition of a letter indicating the SCC*mec* subtype (e.g., ST80-IVc) (1).

**FIG 1:**
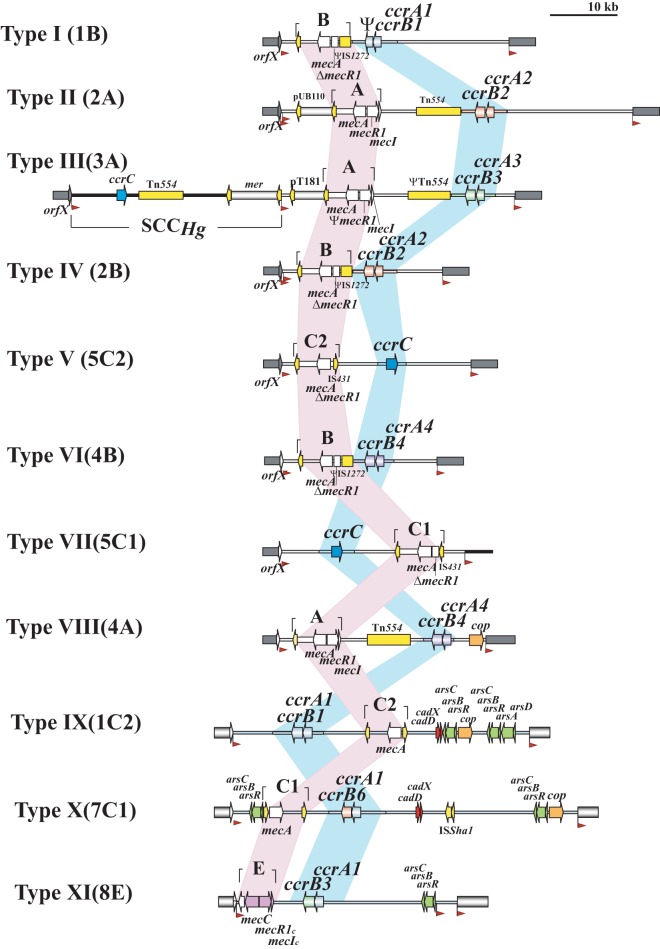
Backbone structure organization of SCC*mec* elements in *S. aureus*. This figure was published previously (4) and is shown here with the kind permission of Keiichi Hiramatsu.

The key components of SCC*mec* are the *mec* and *ccr* gene complexes, which contain genes responsible for methicillin resistance and the mobility of the SCC*mec* element, respectively (2). These two gene complexes are surrounded by three highly variable joining regions (J1 to J3), which contain nonessential components of the SCC*mec* element but may harbor additional resistance genes (3). The currently known SCC*mec* elements of *S. aureus* all integrate at the same unique site in an open reading frame called *orfX*, and all have the same backbone structure with the general organization *orfX*-J3-*mec*-J2*-ccr*-J1, with the exception of SCC*mec* types VIII (4A) and IX (1C2), which have a similar organization, namely, *orfX*-J3-*ccr*-J2-*mec*-J1, as shown in Fig. 1, which was previously published and kindly allowed to be used here by Hiramatsu et al. (4). The combination of the *mec* and *ccr* gene complexes determines the SCC*mec* type, while the J1 region is used for subtyping (2). Historically, subtyping of the SCC*mec* element is only conducted for SCC*mec* types IV (2B) and V (5C2 and 5C2&5).

Genetic rearrangement of the SCC*mec* element can, however, result in novel elements, variants of existing SCC*mec* elements, and composite elements, hence complicating the nomenclature of SCC*mec* elements.

In 2009, the International Working Group on the Classification of Staphylococcal Cassette Chromosome Elements (IWG-SCC) established a consensus uniform nomenclature for the SCC*mec* element that defined the requirements for the annotation of new SCC*mec* elements and provided a homepage for keeping track of approved SCC*mec* elements and information about them. Currently, SCC*mec* types I to XI are listed on the homepage (http://www.sccmec.org), while SCC*mec* types XII (9C2) (5) and XIII (9A) have been reported and approved by the IWG-SCC but yet not listed on the homepage.

The SCC*mec* typing schemes are based on several multiplex PCR (M-PCR) protocols that do not encompass the plasticity of SCC*mec* elements, leading to an increasing proportion of nontypeable (NT) SCC*mec* elements (6–8). Recent advances in whole-genome sequencing (WGS) have made data essential for SCC*mec* typing available, but no bioinformatic tools for *in silico* typing of the SCC*mec* element have been available. Here, we introduce SCC*mec*Finder, an *in silico* web-based bioinformatic tool for the identification and typing of SCC*mec* elements listed by the IWG-SCC from WGS data on *S. aureus* isolates. Together with other existing tools for multilocus sequence typing (MLST) and *spa* typing, SCC*mec*Finder enables high-throughput WGS-based typing of MRSA isolates. The tool is freely available at the Center for Genomic Epidemiology (CGE) homepage, http://genomicepidemiology.org/.

## RESULTS AND DISCUSSION

### SCC*mec* typing by SCC*mec*Finder.

The initial evaluation with the type I to XI reference SCC*mec* elements, the composite elements SCC*mec* types IV (2B&5) and V (5C2&5), subtypes of SCC*mec* types IV (2B) and V (5C2), and the two new elements SCC*mec* types XII (9C2) and XIII (9A) revealed concordant results for all 36 SCC*mec* elements. When both SCC*mec* typing methods (SCC*mec*Finder and the M-PCR-based typing) were applied to the 93 clinical isolates (Table 1), an initial concordance of 96.7% was observed (Table 1). SCC*mec*Finder correctly differentiated methicillin-susceptible *S. aureus* (MSSA) isolates from MRSA isolates in all cases. Of the 50 MSSA isolates, 3 were predicted to harbor an SCC-like element having only the type 1 *ccr* gene complex by SCC*mec*Finder. M-PCR-based typing confirmed the presence of a type 1* ccr* gene complex and the absence of the *mec* gene and gene complex. All three isolates belonged to ST1/t127, which has previously been associated with the SCC-like element SCC*fus*, which contains the fusidic acid resistance gene *fusC* (9). The presence of the *fusC* gene in the three isolates was confirmed, indicating that the isolates harbor the SCC*fus* element. SCC*mec*Finder correctly predicted the SCC*mec* elements in 39 of the 43 MRSA isolates, with an average (*k*-mer) template coverage 83.83%, ranging from 44.12 to 99.79% for the 39 SCC*mec* elements.

**TABLE 1:** SCC*mec* typing results and ST/*spa* type obtained by the three SCC*mec* typing methods for the MRSA strains from the clinical data set

**Strain**	**ST/*spa* type**	**SCC*mec* typing result**	
		PCR-based protocol^a^	**SCC*mec*Finder**
75013	ST30/t019	NT	IV (2B)
75015	ST22/t032	IV (2B)	IV (2B)
75019	ST5/t002	IV (2B)	IV (2B)
75020	ST225/t014	NT	II (2A)
75024	ST152/t355	V (5C2)	NT
75049	ST398/t034	NT	V (5C2&5)
75050	ST30/t019	IV (2B)	IV (2B)
75052	ST87/t216	IV (2B)	IV (2B)
75054	ST398/t034	V (5C2)	V (5C2&5)
75055	ST30/t1752	IV (2B)	IV (2B)
75056	ST8/t008	IV (2B)	IV (2B)
75058	ST6/t4403	NT	IV (2B)
75061	ST30/t019	IV (2B)	IV (2B)
75083	ST22/t3638	IV (2B)	IV (2B&5)
75098	ST5/t1062	IV (2B)	IV (2B)
75100	ST8/t008	IV (2B)	IV (2B)
75101	ST87/t217	IV (2B)	IV (2B)
75103	ST30/t012	IV (2B)	IV (2B)
75104	ST5/t5530	I (1B)	I (1B)
75108	ST45/t026	IV (2B)	IV (2B)
75125	ST5/t002	IV (2B)	IV (2B)
75126	ST45/t362	IV (2B)	IV (2B)
75127	ST45/t362	IV (2B)	IV (2B)
75129	ST22/t022	IV (2B)	IV (2B)
75146	ST398/t034	V (5C2)	V (5C2&5)
75147	ST1/t127	IV (2B)	IV (2B)
75149	ST22/t223	IV (2B)	NT
75150	ST45/t026	IV (2B)	IV (2B)
75178	ST772/t657	V (5C2)	NT
75180	ST5/t002	IV (2B)	NT
75181	ST30/t019	IV (2B)	IV (2B)
75182	ST5/t002	IV (2B)	IV (2B)
75201	ST30/unknown	IV (2B)	IV (2B)
75203	ST22/t032	IV (2B)	IV (2B)
75205	ST5/t045	II (2A)	II (2A)
75208	ST30/t019	IV (2B)	IV (2B)
75209	ST88/t690	IV (2B)	IV (2B)
75211	ST22/t379	IV (2B)	IV (2B)
75212	ST5/t010	I (1B)	I (1B)
75316	ST22/t022	IV (2B&5)	IV (2B&5)
75319	ST8/t008	IV (2B)	IV (2B)
75336	ST152/t355	V (5C2)	V (5C2&5)
75339	ST398/t011	V (5C2)	V (5C2&5)

a The PCR-based SCC*mec* typing protocol described by Kondo et al. (6).

SCC*mec*Finder and the M-PCR-based typing gave NT results for four isolates but without overlaps. Of the four isolates NT by SCC*mec*Finder, two could be recovered by a reassembly of the raw reads using SPAdes instead of the default assembler, e.g., Velvet, and one SCC*mec* element was recovered by modifying the database to the extended database, while the last isolate could not be recovered (Table 2). Upon modification of the thresholds, the percentage of concordance increased to 98.9%.

**TABLE 2:** Recovery of NT SCC*mec* elements

**Strain**	**ST/*spa* type**	**SCC*mec* typing result**		**Recovery method**	**Final SCC*mec* type**
		PCR-based protocol^a^	**SCC*mec*Finder**		
75024	ST152/t355	V (5C2)	NT	Change from Velvet to SPAdes assembler	V (5C2&5)
75149	ST22/t223	IV (2B)	NT	Change from Velvet to SPAdes assembler	IV (2B)
75178	ST772/t657	V (5C2)	V (5C2) or V (5C2&5)	Change from reference to extended database	V (5C2)
75180	ST5/t002	IV (2B)	IV (2B) or II (2A)		NT

a The PCR-based SCC*mec* typing protocol described by Kondo et al. (6).

Publically available *S. aureus* genomes from the NCBI RefSeq database (*n* = 6,852) were included to evaluate the performance of SCC*mec*Finder on genomes with different sequencing qualities from different platforms and sources to catch possible systematic errors by SCC*mec*Finder and identify the most prevalent warning messages. SCC*mec*Finder identified 6,021 genomes as MRSA and 831 as MSSA (Table 3). Less than 8% of the predictions were accompanied by a warning. The predominant warning was the detection of multiple (more than one) *ccr* gene complexes and does not count for SCC*mec* types V (5C2&5) and IV (2B&5), as these are detectable by SCC*mec*Finder. Detection of multiple gene complexes complicates the Basic Local Alignment Search Tool (BLAST)-based prediction, as the method is unable to resolve which gene complexes make up the SCC*mec* element and which may be part of an SCC-like element, and hence, in such cases, SCC*mec*Finder reports the detection of multiple gene complexes to alert the user to make a final decision. Failure of the BLAST-based approach to give a definitive SCC*mec* prediction was also among the most frequently observed warnings, often because of stringent threshold levels or insufficient assembly of contigs. SCC*mec*Finder always detected SCC*mec* types VII (5C1) and X (7C1) by only one of the approaches, which could be indicative of a systematic error by SCC*mec*Finder. However, further investigation revealed no systematic errors.

**TABLE 3:** SCC*mec* typing results produced by SCC*mec*Finder for *S. aureus* genomes available from the NCBI RefSeq database

*S. aureus* group and SCC*mec* typing result	No. of genomes	
	Default thresholds^a^	Modified thresholds^b^
MRSA		
No alerts		
SCC*mec* type I (1B)	201	201
SCC*mec* type II (2A)	2,241	2,241
SCC*mec* type III (3A)	136	136
SCC*mec* type IV (2B)	2,616	2,617
SCC*mec* type IV (2B&5)	20	20
SCC*mec* type V (5C2)	0	34
SCC*mec* type V (5C2&5)	341	342
SCC*mec* type VI (4B)	3	3
SCC*mec* type VII (5C1)	0	0
SCC*mec* type VIII (4A)	5	5
SCC*mec* type IX (1C2)	0	0
SCC*mec* type X (7C1)	0	0
SCC*mec* type XI (8E)	8	8
Alerts		
Multiple complexes		
*ccr* type 1, *ccr* type 2, *mec* class B	8	8
*ccr* type 1, *ccr* type 4, *ccr* type 5, *ccr* type 5, *mec* class C2	1	1
*ccr* type 2, *ccr* type 4, *mec* class A	168^c^	168^c^
*ccr* type 2, *ccr* type 4, *mec* class B	11	11
*ccr* type 2, *ccr* type 4, *ccr* type 5, *mec* class A	8	8
Contradictory prediction at:		
Type level	76	67
Subtype level	76	76
Failure of BLAST	73	73
Failure of *k*-mer	27	1
SCC-like	2	2
MSSA		
No alerts, no SCC*mec* element	773	773
Alerts		
Failure of BLAST	35	34
SCC-like	23	23

a In all, 6,021 MRSA and 831 MSSA isolates were tested.

b The modifications included lowering the thresholds to >50% nucleotide identity and >50% template coverage and changing from the reference database to the extended database. In all, 6,022 MRSA and 830 MSSA isolates were tested.

c A total of 147/168 strains causing alerts belonged to the same ST (ST5), indicating a single NT element.

### SCC*mec* subtype prediction by SCC*mec*Finder.

The subtyping predictions by SCC*mec*Finder of SCC*mec* types IV (2B) and V (5C2) were initially evaluated by using the reference elements listed by the IWG-SCC. The evaluation showed perfect concordance. The subtyping predictions were further evaluated by comparing the findings of SCC*mec*Finder with those of PCR-based SCC*mec* typing (Table 4). Sorting on the basis of the ST type, *spa* type, and subtype predicted by SCC*mec*Finder revealed a clustering of the isolates. Comparing the clustering results with the expected subtype prediction based on the multilocus ST, the *spa* type, a literature search, and PCR-based subtyping revealed a high concordance of the subtyping predictions, indicating that the SCC*mec* (sub)types are relatively stable within the ST/*spa* type clone (Table 4).

**TABLE 4:** Comparison of the subtype predictions of SCC*mec* types IV (2B) and V (5C2) from the clinical data set by the three SCC*mec* typing methods

**Strain**	**ST/*spa* type**	**SCC*mec* typing result**	
		PCR-based protocol^a^	**SCC*mec*Finder**
75178	ST772/t657	NT	Va (5C2)
75024	ST152/t355	NT	Vnt (5C2&5)
75336	ST152/t355	NT	Vc (5C2&5)
75054	ST398/t034	NT	Vc (5C2&5)
75339	ST398/t011	NT	Vc (5C2&5)
75049	ST398/t034	NT	Vc (5C2&5)
75013	ST30/t019	IVc (2B)	IVc (2B)
75050	ST30/t019	IVc (2B)	IVc (2B)
75055	ST30/t1752	IVc (2B)	IVc (2B)
75061	ST30/t019	IVc (2B)	IVc (2B)
75181	ST5/t002	IVc (2B)	IVc (2B)
75208	ST30/t019	IVc (2B)	IVc (2B)
75108	ST45/t026	IVa (2B)	IVa (2B)
75150	ST45/t026	IVa (2B)	IVa (2B)
75126	ST45/t362	IVb (2B)	IVb (2B)
75127	ST45/t362	IVb (2B)	IVb (2B)
75052	ST87/t216	NT	IVg (2B)
75101	ST87/t216	NT	IVg (2B)
75015	ST22/t032	NT	IVnt (2B)^b^
75129	ST22/t022	NT	IVnt (2B)^b^
75203	ST22/t032	NT	IVnt (2B)^b^
75211	ST22/t379	NT	IVnt (2B)^b^
75098	ST5/t1062	IVc (2B)	IVc (2B)
75180	ST5/t002	IVc (2B)	IVc (2B)
75019	ST5/t002	IVc (2B)	IVc (2B)
75125	ST5/t002	IVc (2B)	IVc (2B)
75182	ST5/t002	IVc (2B)	IVc (2B)
75201	ST30/t019	IVc (2B)	IVc (2B)
75103	ST30/t012	IVc (2B)	IVc (2B)
75319	ST8/t008	IVa (2B)	IVa (2B)
75056	ST8/t008	IVc (2B)	IVc (2B)
75100	ST8/t008	NT	IVnt (2B)^c^
75147	ST1/t127	IVa (2B)	IVa (2B)
75058	ST6/t4403	IVa (2B)	IVa (2B)
75209	ST88/t690	IVa (2B)	IVa (2B)

a The PCR-based SCC*mec* typing protocol described by Kondo et al. (6).

b Subtype either IVh (2B) or IVj (2B).

c Contradictory subtype prediction.

### Database curation and identification of novel (sub)types.

SCC*mec*Finder is designed to detect all of the reference SCC*mec* types listed by the IWG-SCC, and ideally, novel SCC*mec* (sub)types will be added to the databases by the curator after agreement with the IWG-SCC group upon the detection of a novel SCC*mec* (sub)type. Likewise, we suggest that notification of the IWG-SCC should be reported in the article describing the element.

The combination of two different approaches for the prediction of the SCC*mec* element in SCC*mec*Finder creates an opportunity for the user to assess whether the SCC*mec* element in question is a variant of an existing element or a novel element, as SCC*mec*Finder provides data on the detected target genes and the *k*-mer template coverage. An example is *S. aureus* JCSC7481 (GenBank accession no. AB774378), which the authors have stated is a variant of SCC*mec* type V (5C2). Running the SCC*mec* element of *S. aureus* JCSC7481 through SCC*mec*Finder by using the reference database, an alert about contradictory predictions was produced. The *k*-mer template coverage was 42.4%, indicating that the element differed from the reference elements and in concordance with the conclusion of the authors that it was a variant of SCC*mec* type V (5C2). However, the element of *S. aureus* JCSC7481 has, to our knowledge, not yet been assigned by the IWG-SCC, and hence, the SCC*mec* element has not yet been added to the reference database (but is currently found in the extended database). Of importance; researchers are encouraged to consult members of the IWG-SCC and the curator of the SCC*mec*Finder database to keep the number of NT SCC*mec* elements low.

### Conclusion.

In conclusion, SCC*mec*Finder (https://cge.cbs.dtu.dk/services/SCCmecFinder) is a validated tool for rapid whole-genome-based SCC*mec* typing of MRSA that, together with the existing tools for the *in silico* prediction of the multilocus ST (https://cge.cbs.dtu.dk/services/MLST) and *spa* type (https://cge.cbs.dtu.dk/services/spatyper), can provide a standardized nomenclature of *S. aureus* genomes. It is our intention that the SCC*mec*Finder database will be curated in a way that fulfills future needs to encompass the changing epidemiology of MRSA accompanied by increasing genetic diversity.

## MATERIALS AND METHODS

### Construction of SCC*mec*Finder.

**FIG 2:**
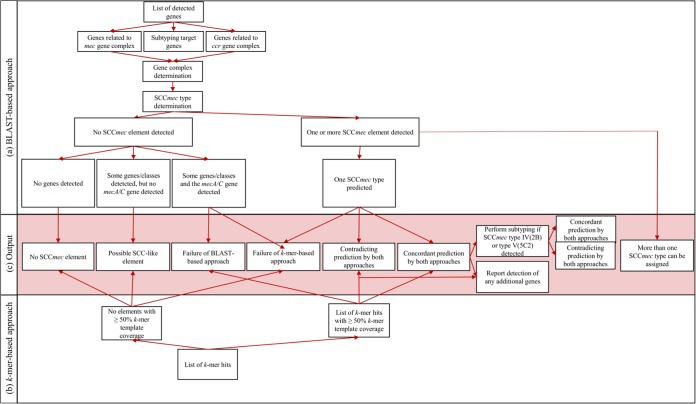
Workflow describing the decision-making of SCC*mec*Finder. The output (c) combines interpretations from the BLAST (a)- and *k*-mer (b)-based approaches. The output can be no, one, or more than one SCC*mec* element detected.

SCC*mec*Finder combines two existing and validated gene prediction algorithms (10, 11), a BLAST-based approach and a *k*-mer-based approach. By utilizing the BLAST approach (BLASTn algorithm) with default thresholds set at ≥90% nucleotide identity and a minimum length of 60%, SCC*mec*Finder detects the presence of selected target genes, identifies the *mec* and *ccr* gene complex, and types the SCC*mec* element, whereas by utilizing the *k*-mer-approach, SCC*mec*Finder types the element on the basis of homology to reference SCC*mec* elements by identifying the number of co-occurring DNA sequences with the length *k*, known as *k*-mers, between the query genome and the entries in the curated database, of which only hits with ≥50% *k*-mer template coverage are considered (Fig. 2). Less stringent thresholds for the BLAST-based approach can be set, but less stringent thresholds could lead to outputs that require more downstream analysis and might give misleading annotations. The software can be found at https://bitbucket.org/genomicepidemiology/sccmecfinder.git. Prior for choosing the thresholds for each algorithm, a parameterization study was conducted.

SCC*mec*Finder compares the predicted SCC*mec* types from the BLAST- and the *k*-mer-based predictions and checks for contradictory results to achieve correct SCC*mec* annotation. Identification of SCC*mec* type IV (2B) or V (5C2) results in subtyping of the element. Subtyping is performed as with the typing of the SCC*mec* element, on the basis of a BLAST search for selected subtyping target genes from the J1 region and the *k*-mer-based approach. On the basis of the comparison, SCC*mec*Finder outputs one or more of the five possible output types, e.g., notification of a positive/negative prediction, a contradictory SCC*mec* type/subtype, a missing/additional gene(s), the detection of multiple gene complexes, or the presence of a possible SCC-like element. If one of the prediction approaches fails, the output will be negative but will contain information about the possible presence of an SCC*mec* element to guide the user to perform further analysis (Fig. 2).

### Sequence databases for SCC*mec*Finder.

Two databases were developed to support the two different approaches. For the BLAST-based approach, a collection of 37 target gene sequences (Table S1 and S2) and for the *k*-mer-based approach, a collection of 36 archetypal reference SCC*mec* were selected to represent sequences of known types available at/according to the IWG-SCC (Table S3). Additionally, an extended version of the database for the *k*-mer-based approach, containing all of the archetypal reference SCC*mec* elements and variants of SCC*mec* type V (5C2 and 5C2&5) from *S. aureus* that have not yet been validated or assigned a subtype by the IWG-SCC, was also generated (Table S4). Each entry in all three databases is accompanied by an NCBI GenBank accession number, thus allowing for the correlation of an identified gene(s)/element(s) in the query genome with an annotated gene(s)/element(s) in the archetypal reference SCC*mec* elements. The databases for SCC*mec*Finder can be found at https://bitbucket.org/genomicepidemiology/sccmecfinder_db.git.

10.1128/mSphere.00612-17.1 TABLE S1  Target genes included in the database and used for detection of the *mec* and *ccr* gene complexes for BLAST-based SCC*mec* typing. Download TABLE S1, PDF file, 0.1 MB. Copyright © 2018 Kaya et al. 2018 Kaya et al. This content is distributed under the terms of the Creative Commons Attribution 4.0 International license.

10.1128/mSphere.00612-17.2 TABLE S2  Subtype target genes included in the database for the BLAST-based approach and used for SCC*mec* types IV (2B) and V (5C2 and 5C2&5) in BLAST-based SCC*mec* typing. Download TABLE S2, PDF file, 0.1 MB. Copyright © 2018 Kaya et al. 2018 Kaya et al. This content is distributed under the terms of the Creative Commons Attribution 4.0 International license.

10.1128/mSphere.00612-17.3 TABLE S3  SCC*mec* elements included in the reference database used for the *k*-mer-based approach. Download TABLE S3, PDF file, 0.1 MB. Copyright © 2018 Kaya et al. 2018 Kaya et al. This content is distributed under the terms of the Creative Commons Attribution 4.0 International license.

10.1128/mSphere.00612-17.4 TABLE S4  SCC*mec* elements included in the extended database used for the *k*-mer-based approach. Download TABLE S4, PDF file, 0.1 MB. Copyright © 2018 Kaya et al. 2018 Kaya et al. This content is distributed under the terms of the Creative Commons Attribution 4.0 International license.

### User interface and use of SCC*mec*Finder.

SCC*mec*Finder builds on the common design of the CGE user interface. The upload page consists of four dropdown menus by which the type of sequencing platform can be chosen and default settings for the analysis can be changed. The output page consists of three sections, with the first reporting the presence/absence of the *mecA*/*mecC* gene. The second section is the actual output of the SCC*mec* prediction, in which the *mec* and *ccr* gene complexes, SCC*mec* type, and possible subtype from both approaches are reported. The third and last section is reporting of the genes detected by the BLAST-based approach and the top five hits from the *k*-mer-based approach.

Submission to SCC*mec*Finder can be done by using either preassembled draft genomes or raw reads from the 454, Ion Torrent, or Illumina sequencing platform. Prior to the BLAST-based prediction, 454 and Ion Torrent raw reads are *de novo* assembled by using Newbler, while Illumina raw reads are *de novo* assembled by using Velvet (10).

### SCC*mec* typing using SCC*mec*Finder and validation.

The integrity of SCC*mec*Finder was initially evaluated by submitting reference genomes and the archetypal SCC*mec* elements included in the *k*-mer reference database by using the default thresholds. After the initial validation process, draft genomes of 93 clinical isolates were submitted to SCC*mec*Finder without further processing.

The isolates covered a diverse collection of 43 MRSA and 50 MSSA strains representing 13 different STs of predominant MRSA and MSSA clones in Denmark. The genomes were obtained after genomic DNA extraction (DNeasy Blood and Tissue kit; Qiagen, Copenhagen, Denmark), and fragment libraries were constructed by using an in-house library protocol (12), followed by 100-bp paired-end sequencing (HiSeq; Illumina) in accordance with the manufacturer’s instructions. *De novo* assembly was performed with the modified Velvet algorithm integrated into the web tool Assembler v1.2 (available at https://cge.cbs.dtu.dk/services/Assembler/). The *in silico* predictions by SCC*mec*Finder were evaluated by comparing the results with those predicted by the PCR-based protocol of Kondo et al. (6), M-PCRs 1 and 2. Subtyping were performed by M-PCR 3 as described by Kondo et al. (6). Composite elements were detected by ensuring the presence of both *ccr* gene complexes in SCC*mec* types IV (2B&5) and V (5C2&5), and an in-house protocol was used for the typing of *ccr*C1 gene alleles 2 and 8, respectively, for the verification of SCC*mec* type V (5C2&5) (13).

### Additional genomes.

Finally, 6,852 publically available *S. aureus* draft genomes from the NCBI Reference Sequence Database (RefSeq [https://ncbi.nlm.nih.gov/refseq/], accessed 16 September 2016) were downloaded to validate the SCC*mec*Finder outputs for any systematic errors. The assemblies were subjected to rough quality filtering on the basis of being *S. aureus* (determined on the basis of MLST and single nucleotide polymorphism and phylogenetic analyses), their genome size, number of contigs, and *N*_50_ value, where genomes with a size of <2.5 Mb, an *N*_50_ value of <10,000, and >1,000 contigs were discarded. All genomes were subjected to MLST (http://github.com/tseemann/mlst) and subsequently submitted to SCC*mec*Finder by using default thresholds.

### Accession number(s).

All of clinical sequences included in this study are available at the European Nucleotide Archive under project accession number PRJEB22380.
