# Supplementary material for: SCCmecFinder, a Web-Based Tool for Typing of Staphylococcal Cassette Chromosome mec in Staphylococcus aureus Using Whole-Genome Sequence Data
Source: mSphere. 2018 Feb 14;3(1):e00612-17. doi: 10.1128/mSphere.00612-17 (PMC5812897; doi:10.1128/mSphere.00612-17)
Supplement: TABLE S1 [file sph001182472st1.pdf]

TABLE S1

Gene complexes	Target gene/complex
mec gene complex	*mecA*
	mecI
	mecR1
	Δ*mecR1*
	IS*1272*
	mecA-Δ*mecR1*-IS*431*[a]
	mecA-Δ*mecR1*-IS*431*[b]
	$mecA_{LGA251}$
ccr gene complex	*ccrA1*
	ccrA2
	ccrA3
	ccrA4
	ccrA5
	ccrB1
	ccrB2
	ccrB3
	ccrB4
	ccrB6
	ccrC1 allele *1*
	ccrC1 allele *2*
	ccrC1 allele *3*
	ccrC1 allele *4*
	ccrC1 allele *5*[c]
	ccrC1 allele *6*[c]
	ccrC1 allele *7*[d]
	ccrC1 allele *8*

[a] Partial *mec* class C1 gene complex
[b] Partial *mec* class C2 gene complex
[c] *Staphylococcus haemolyticus*
[d] *Staphylococcus epidermidis*
