# Supplementary material for: SCCmecFinder, a Web-Based Tool for Typing of Staphylococcal Cassette Chromosome mec in Staphylococcus aureus Using Whole-Genome Sequence Data
Source: mSphere. 2018 Feb 14;3(1):e00612-17. doi: 10.1128/mSphere.00612-17 (PMC5812897; doi:10.1128/mSphere.00612-17)
Supplement: TABLE S2 [file sph001182472st2.pdf]

TABLE S2

SCC*mec* (sub)type	Target gene	Strain	Size (bp)	GenBank accession no.	Reference
SCC*mec* type IVa (2B)	CQ002	CA05	1491	AB063172	(1)
SCC*mec* type IVb (2B)	M001	8/6-3P	1431	AB063173	(1)
SCC*mec* type IVc (2B)	CR008	81/108	1155	AB096217	(1)
SCC*mec* type IVd (2B)	CG002	JCSC4469	1872	AB097677	(1)
SCC*mec* type IVg (2B)	PK02	M03-68	882	DQ106887	(2)
SCC*mec* type IVh (2B)	ER16_00235	H-EMRSA-15	378	CP007659	(3)
SCC*mec* type IVi (2B)	CB18[a]	JCSC6668	1446	AB425823	(4)
SCC*mec* type IVj (2B)	C20	JCSC6670	498	AB425824	(5)
SCC*mec* type Va (5C2)	V024	WIS	813	AB121219	(1)
SCC*mec* type Vb (5C2&5)	ORF No.33	JCSC5952	1302	AB462393	(2)
SCC*mec* type Vc (5C2&5)	*czrC*[b]	JCSC6944	1935	AB505629	(6)

[a] Defined by this study
[b] Termed *copA* in GenBank

References

1. Kondo Y, Ito T, Ma XX, Watanabe S, Kreiswirth BN, Etienne J, Hiramatsu K. 2007. Combination of multiplex PCRs for staphylococcal cassette chromosome *mec* type assignment: Rapid identification system for *mec*, *ccr*, and major differences in junkyard regions. Antimicrob Agents Chemother 51:264–274.

2. Milheiriço C, Oliveira DC, de Lencastre H. 2007. Multiplex PCR strategy for subtyping the staphylococcal cassette chromosome *mec* type IV in methicillin-resistant *Staphylococcus aureus*: “SCC*mec* IV multiplex.” J Antimicrob Chemother 60:42–48.

3. Ito T, Hiramatsu K, Oliveira DC, De Lencastre H, Zhang K, Westh H, O’Brien F, Giffard PM, Coleman D, Tenover FC, Boyle-Vavra S, Skov RL, Enright MC, Kreiswirth B, Kwan SK, Grundmann H, Laurent F, Sollid JE, Kearns AM, Goering R, John JF, Daum R, Soderquist B. 2009. Classification of staphylococcal cassette chromosome *mec* (SCC*mec*): Guidelines for reporting novel SCC*mec* elements. Antimicrob Agents Chemother 53:4961–4967.

4. Berglund C, Ito T, Ikeda M, Xiao XM, Söderquist B, Hiramatsu K. 2008. Novel type of staphylococcal cassette chromosome *mec* in a methicillin-resistant *Staphylococcus aureus* strain isolated in Sweden. Antimicrob Agents Chemother 52:3512–3516.

5. Berglund C, Ito T, Ma Xiao Xue XX, Ikeda M, Watanabe S, Söderquist B, Hiramatsu K. 2009. Genetic diversity of methicillin-resistant *Staphylococcus aureus* carrying type IV SCC*mec* in Örebro County and the western region of Sweden. J Antimicrob Chemother 63:32–41.

6. Li S, Skov RL, Han X, Larsen AR, Larsen J, Sørum M, Wulf M, Voss A, Hiramatsu K, Ito T. 2011. Novel types of staphylococcal cassette chromosome *mec* elements identified in clonal complex 398 methicillin-resistant *Staphylococcus aureus* strains. Antimicrob Agents Chemother 55:3046–3050.
