# Supplementary material for: SCCmecFinder, a Web-Based Tool for Typing of Staphylococcal Cassette Chromosome mec in Staphylococcus aureus Using Whole-Genome Sequence Data
Source: mSphere. 2018 Feb 14;3(1):e00612-17. doi: 10.1128/mSphere.00612-17 (PMC5812897; doi:10.1128/mSphere.00612-17)
Supplement: TABLE S3 [file sph001182472st3.pdf]

TABLE S3. List of SCCmec elements included in the reference database for the *k*-mer-based approach.

SCC*mec* (sub)type	Strain	GenBank accession no.	Reference
SCC*mec* type I (1B)	NCTC10442	AB033763.2	(1)
	COL	CP000046	(1)
SCC*mec* type II (2A)	N315	D86934.2	(1)
	Mu50	BA000017	(1)
	Mu3	AP009324	(1)
	MRSA252	BX571856	(1)
	JH1	CP000737	(1)
	JH9	CP000703	(1)
SCC*mec* type III (3A)	85/2082	AB037671	(1)
SCC*mec* type IVa (2B)	CA05	AB063172	(1)
	MW2	BA000033	(1)
SCC*mec* type IVb (2B)	8/6-3P	AB063173	(1)
SCC*mec* type IVc (2B)	81/108	AB096217	(1)
	AR43/3330.1	AJ810121	(1)
	2314	AY271717	(1)
	cm11	EU437549	(1)
SCC*mec* type IVd (2B)	JCSC4469	AB097677[a]	(1)
	BK2529	NZ_JYBA01000013	
SCC*mec* type IVg (2B)	M03-68	DQ106887	(1)
SCC*mec* type IVh (2B)	H-EMRSA-15	CP007659	(1)
SCC*mec* type IVi (2B)	JCSC6668	AB425823	(1)
SCC*mec* type IVj (2B)	JCSC6670	AB425824	(1)
SCC*mec* type IVF(2B)[b]	Not available	Not available	(2)
SCC*mec* type IV (2B&5)	ZH47	AM292304	(1)
SCC*mec* type Va (5C2)	WIS	AB121219	(1)
SCC*mec* type Vb (5C2&5)	PM1	AB462393	(1)
	TSGH17	AB512767	(1)
	JCSC5952	AB478780	(3)
SCC*mec* type Vc (5C2&5)	JCSC6944	AB505629	(3)
SCC*mec* type VI (4B)	HDE288	AF411935	(1)
SCC*mec* type VII (5C1)	JCSC6082	AB373032	(1)
SCC*mec* type VIII (4A)	C10682	FJ390057	(1)
	BK20781	FJ670542	(1)
SCC*mec* type IX (1C2)	JCSC6943	AB505628	(1)
SCC*mec* type X (7C1)	JCSC6945	AB505630	(1)
SCC*mec* type XI (8E)	LGA251	FR821779	(1)
SCC*mec* type XII (9C2)	BA01611	KR187111	

[a] Partial sequence
[b] Not included in SCC*mec*Finder

References

1. Ito T, Hiramatsu K, Oliveira DC, De Lencastre H, Zhang K, Westh H, O'Brien F, Giffard PM, Coleman D, Tenover FC, Boyle-Vavra S, Skov RL, Enright MC, Kreiswirth B, Kwan SK, Grundmann H, Laurent F, Sollid

JE, Kearns AM, Goering R, John JF, Daum R, Soderquist B. 2009. Classification of staphylococcal cassette chromosome *mec* (SCC*mec*): Guidelines for reporting novel SCC*mec* elements. Antimicrob Agents Chemother 53:4961–4967.

2. Shore A, Rossney AS, Keane CT, Enright MC, Coleman DC. 2005. Seven novel variants of the staphylococcal chromosomal cassette *mec* in methicillin-resistant *Staphylococcus aureus* isolates from Ireland. Antimicrob Agents Chemother 49:2070–2083.

3. Li S, Skov RL, Han X, Larsen AR, Larsen J, Sørum M, Wulf M, Voss A, Hiramatsu K, Ito T. 2011. Novel types of staphylococcal cassette chromosome *mec* elements identified in clonal complex 398 methicillin-resistant *Staphylococcus aureus* strains. Antimicrob Agents Chemother 55:3046–3050.
